# The genome sequence of a darkling beetle, *Lagria hirta *(Linnaeus, 1758)

**DOI:** 10.12688/wellcomeopenres.20232.1

**Published:** 2023-11-01

**Authors:** Olga Sivell, Brian Levey, Maxwell V. L. Barclay, Natural History Museum Genome Acquisition Lab, Darwin Tree of Life Barcoding collective, Wellcome Sanger Institute Tree of Life programme, Wellcome Sanger Institute Scientific Operations: DNA Pipelines collective, Tree of Life Core Informatics collective, Darwin Tree of Life Consortium

**Affiliations:** 1 Natural History Museum, London, England, UK; 2 National Museum of Wales, Cardiff, Wales, UK

**Keywords:** Lagria hirta, darkling beetle, genome sequence, chromosomal, Coleoptera

## Abstract

We present a genome assembly from an individual male *Lagria hirta* (darkling beetle; Arthropoda; Insecta; Coleoptera; Tenebrionidae). The genome sequence is 336.8 megabases in span. Most of the assembly is scaffolded into 12 chromosomal pseudomolecules, including the X and Y sex chromosomes. The mitochondrial genome has also been assembled and is 16.12 kilobases in length. Gene annotation of this assembly on Ensembl identified 12,850protein coding genes.

## Species taxonomy

Eukaryota; Metazoa; Eumetazoa; Bilateria; Protostomia; Ecdysozoa; Panarthropoda; Arthropoda; Mandibulata; Pancrustacea; Hexapoda; Insecta; Dicondylia; Pterygota; Neoptera; Endopterygota; Coleoptera; Polyphaga; Cucujiformia; Tenebrionoidea; Tenebrionidae; Lagriinae; *Lagria*; *Lagria hirta* (Linnaeus, 1758) (NCBI:txid296003).

## Background

*Lagria hirta* (Linnaeus, 1758) is widely distributed in the western Palaearctic (across the whole of Europe, Algeria and Morocco) and in Asia (Russia: Western and Eastern Siberia, Israel, Cyprus, Turkey, Iran, Iraq, Kazakhstan, Turkmenistan, Uzbekistan and Tajikistan) ( Merkl, 2006).

*Lagria hirta* is the only common species of the subfamily Lagriinae (Coleoptera, Tenebrionidae) in Central Europe ( Zhou, 2001), and one of only two species occurring in Britain. They are both elongate beetles, with a black head, pronotum and legs and pubescent elytra that are yellow to dark brown. They are sexually dimorphic, with the males more elongate and parallel sided, the females with the abdomen and elytra broader and more drop shaped. With a body length of 7–10 mm, *L. hirta* is slightly smaller than the very similar but much rarer *Lagria atripes* (10–12 mm) and also has proportionately smaller eyes. L. hirta has a strongly punctured pronotum (smoother and more glabrous in *L. atripes*) and the pubescence of the elytra is simple (arranged in a ‘herring bone pattern’ in *L. atripes*) ( Buck, 1954). *L. atripes* is restricted to a few good quality deciduous forests in southern England.

*Lagria hirta* is crepuscular and nocturnal. The beetles can be encountered on flowers and shrubs. They are polyphagous and feed on young leaves, nectar and pollen ( Hůrka, 2005; Zhou, 2001). They were also reported to cause damage to spruce by feeding on young shoots, but are not of economic importance unlike some closely related species from Africa, Australia and Asia ( Postner, 1961). The brownish larvae are saprophagous ( Zhou, 2001), free living, can be swept from vegetation at night, and have a distinctive appearance with tufts of erect setae on the sides of the abdominal segments, and two small triangular urogomphi at the apex of the abdomen.

This species has an annual life cycle, with adults active from late May or June, peaking in July, and with females sometimes surviving well into September. The eggs are laid in batches of approximately 80 at a time into soil or leaf litter. Unmated females can lay a batch of eggs, but these are not fertile and will not hatch ( Stammer, 1929; Zhou, 2001). In laboratory conditions egg hatching occurred in temperatures of 10–25 °C, while at 5 °C and 30 °C all eggs failed ( Zhou & Topp, 2003). Typically, there are 8-10 larval instars ( Zhou, 1996; Zhou & Topp, 2003). The larva requires a period of cooler temperatures to progress to pupation. The experiments conducted in laboratory conditions showed that larvae reared at constant temperatures and without a chilling period were unable to pupate successfully. Some reached the 9-10 ^th^ instar, hardly any pupated and all subsequently died ( Zhou & Topp, 2003). In natural conditions *L. hirta* overwinters as a larva and pupation occurs in early May ( Postner, 1961).

Throughout their life cycle *Lagria* beetles live in symbiosis with antibiotic producing bacteria dominated by the genus *Burkholderia*. They are transferred from female accessory glands to an egg during oviposition and protect it against pathogenic fungi. Subsequently these bacteria colonize specialized dorsal cuticular structures of the mesothorax, metathorax, and the first abdominal segment in both sexes of larvae. They are absent in adult males ( Flórez, 2016; Flórez & Kaltenpoth, 2017; Flórez *et al.*, 2017; Janke *et al.*, 2022; Stammer, 1929). The study conducted on closely related *L. villosa* showed the beetles can also acquire bacteria from dry leaf litter and in turn bacteria can be transferred from a beetle host to the soil, leaf litter or a plant ( Wierz *et al.*, 2021).

*Lagria hirta* is widely distributed in Britain, common in Wales and England north to South Yorkshire, with scattered records up to north-eastern Scotland ( UK Beetles, no date). It can be found in woodland, at the edges of broadleaved and coniferous forests, coastal scrub and sand dunes, urban parks and gardens, meadows, clearings, near old hedges, on grasses, flowers and shrubs, and can be attracted to light ( Hůrka, 2005; James, 2018). It is the most common and widespread member of the family Tenebrionidae in the United Kingdom.

The high-quality genome sequence described here is the first one reported for *Lagria hirta.* It was sequenced from one male specimen from Penhale Dunes, England. It will aid research on the biology, phylogeny and ecology of this species. The genome has been generated as part of the Darwin Tree of Life Project, a collaborative effort to sequence all named eukaryotic species in the Atlantic Archipelago of Britain and Ireland.

## Genome sequence report

**Figure 1.**
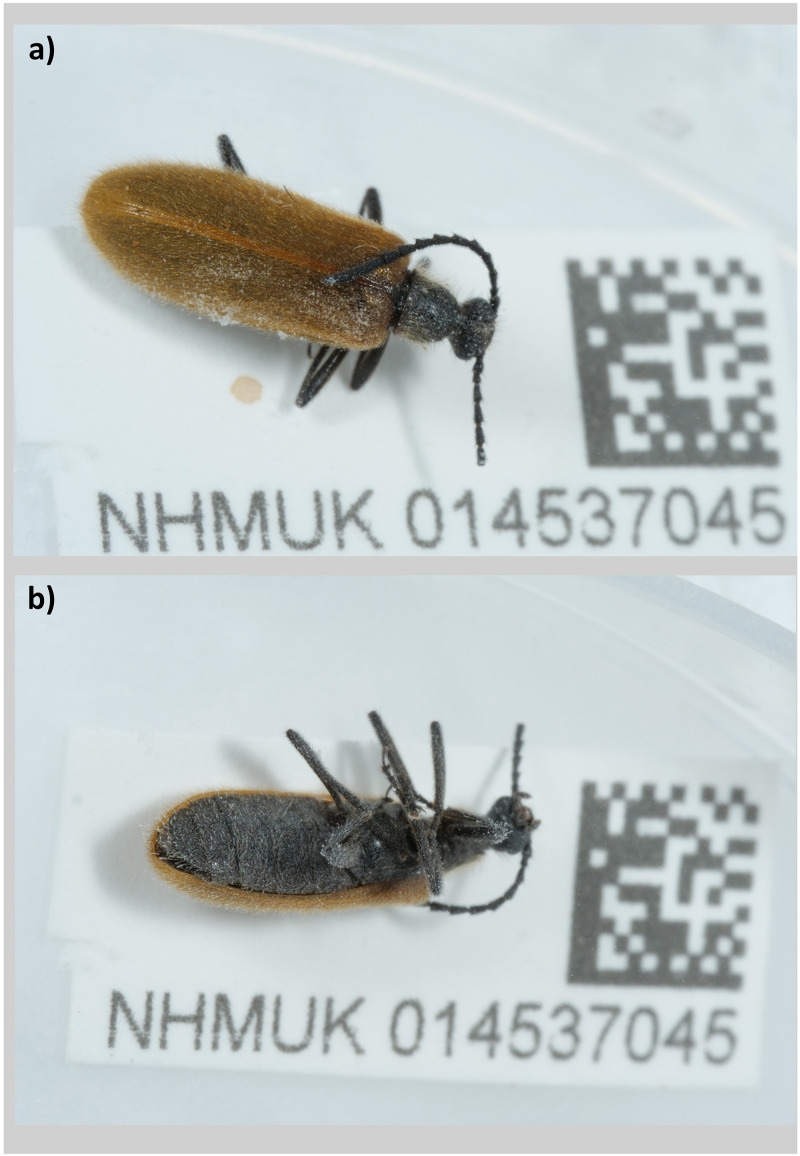
Photograph of the *Lagria hirta* (Linnaeus, 1758) specimen NHMUK014537045 taken during sample preservation and processing. **a**) A habitus of the specimen in dorsal view. **b**) The specimen in ventral view.

The genome was sequenced from one male *Lagria hirta* ( Figure 1) collected from Penhale Dunes, England, UK (50.37, –5.14). A total of 72-fold coverage in Pacific Biosciences single-molecule HiFi long reads was generated. Primary assembly contigs were scaffolded with chromosome conformation Hi-C data. Manual assembly curation corrected 70 missing joins or misjoins and removed 3 haplotypic duplications, reducing the assembly length by 0.25% and the scaffold number by 67.5%, and increasing the scaffold N50 by 4.31%.

**Figure 2.**
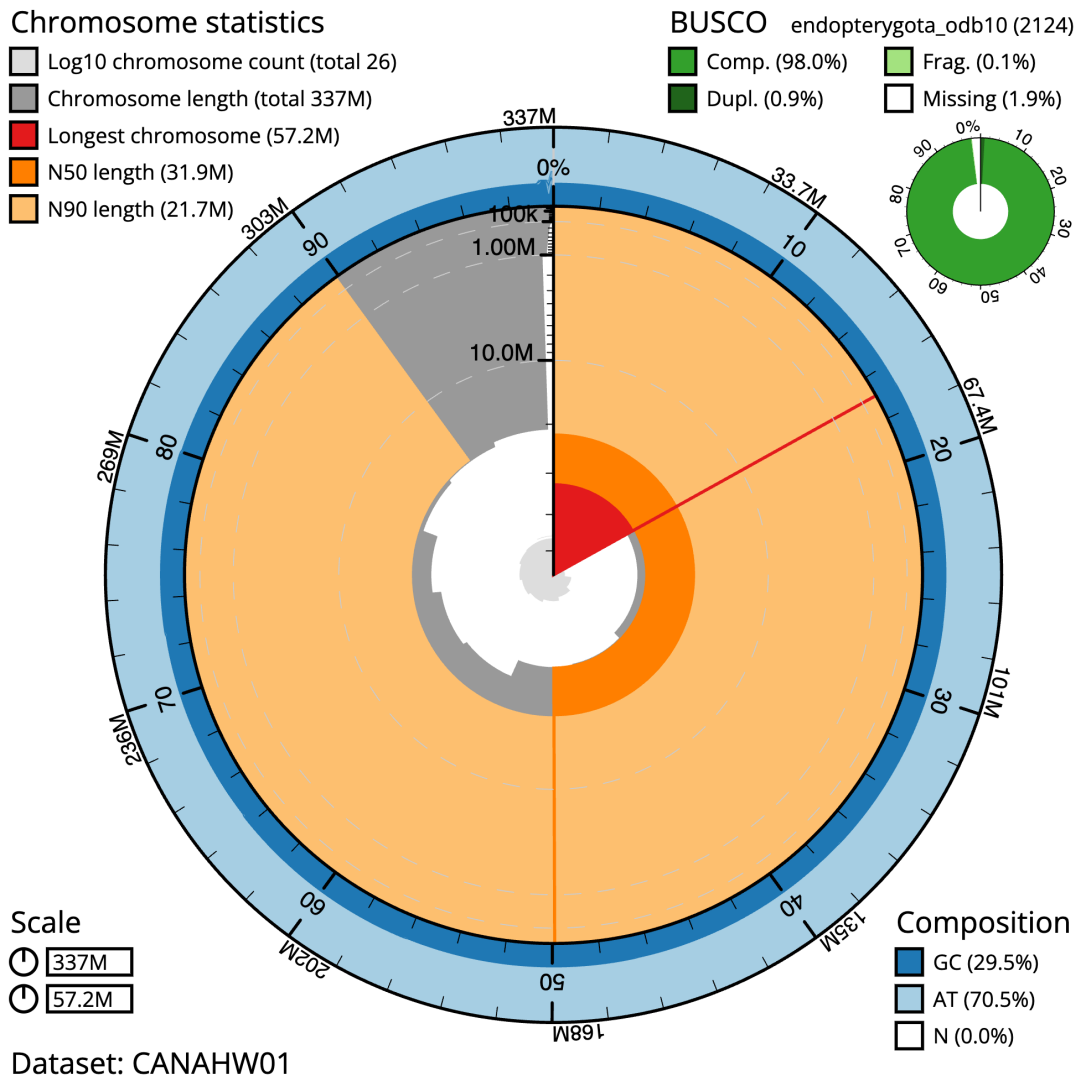
Genome assembly of *Lagria hirta*, icLagHirt1.1: metrics. The BlobToolKit Snailplot shows N50 metrics and BUSCO gene completeness. The main plot is divided into 1,000 size-ordered bins around the circumference with each bin representing 0.1% of the 336,783,501 bp assembly. The distribution of scaffold lengths is shown in dark grey with the plot radius scaled to the longest scaffold present in the assembly (57,187,386 bp, shown in red). Orange and pale-orange arcs show the N50 and N90 scaffold lengths (31,908,519 and 21,721,301 bp), respectively. The pale grey spiral shows the cumulative scaffold count on a log scale with white scale lines showing successive orders of magnitude. The blue and pale-blue area around the outside of the plot shows the distribution of GC, AT and N percentages in the same bins as the inner plot. A summary of complete, fragmented, duplicated and missing BUSCO genes in the endopterygota_odb10 set is shown in the top right. An interactive version of this figure is available at https://blobtoolkit.genomehubs.org/view/Lagria%20hirta/dataset/CANAHW01/snail.

**Figure 3.**
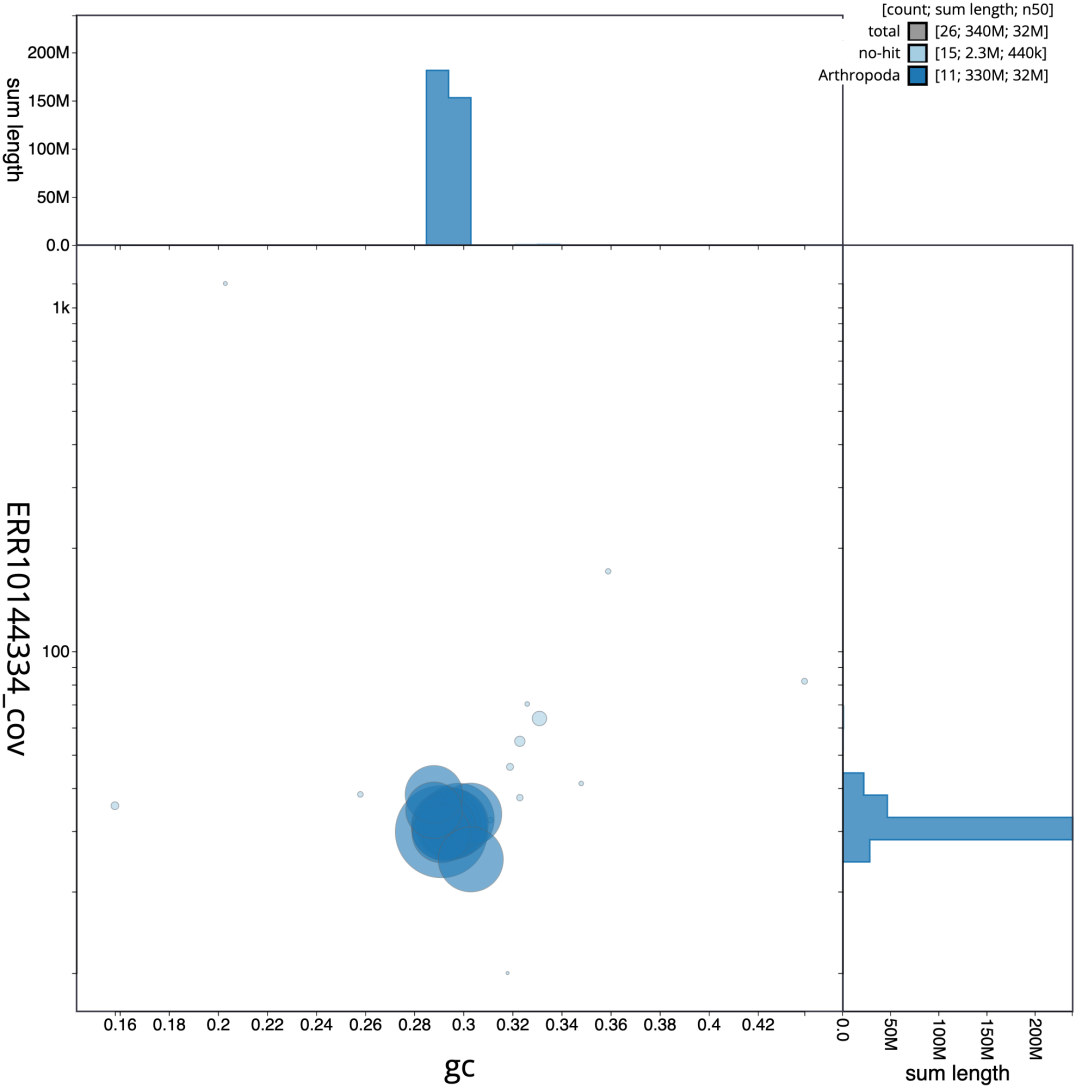
Genome assembly of *Lagria hirta*, icLagHirt1.1: BlobToolKit GC-coverage plot. Scaffolds are coloured by phylum. Circles are sized in proportion to scaffold length. Histograms show the distribution of scaffold length sum along each axis. An interactive version of this figure is available at https://blobtoolkit.genomehubs.org/view/Lagria%20hirta/dataset/CANAHW01/blob.

**Figure 4.**
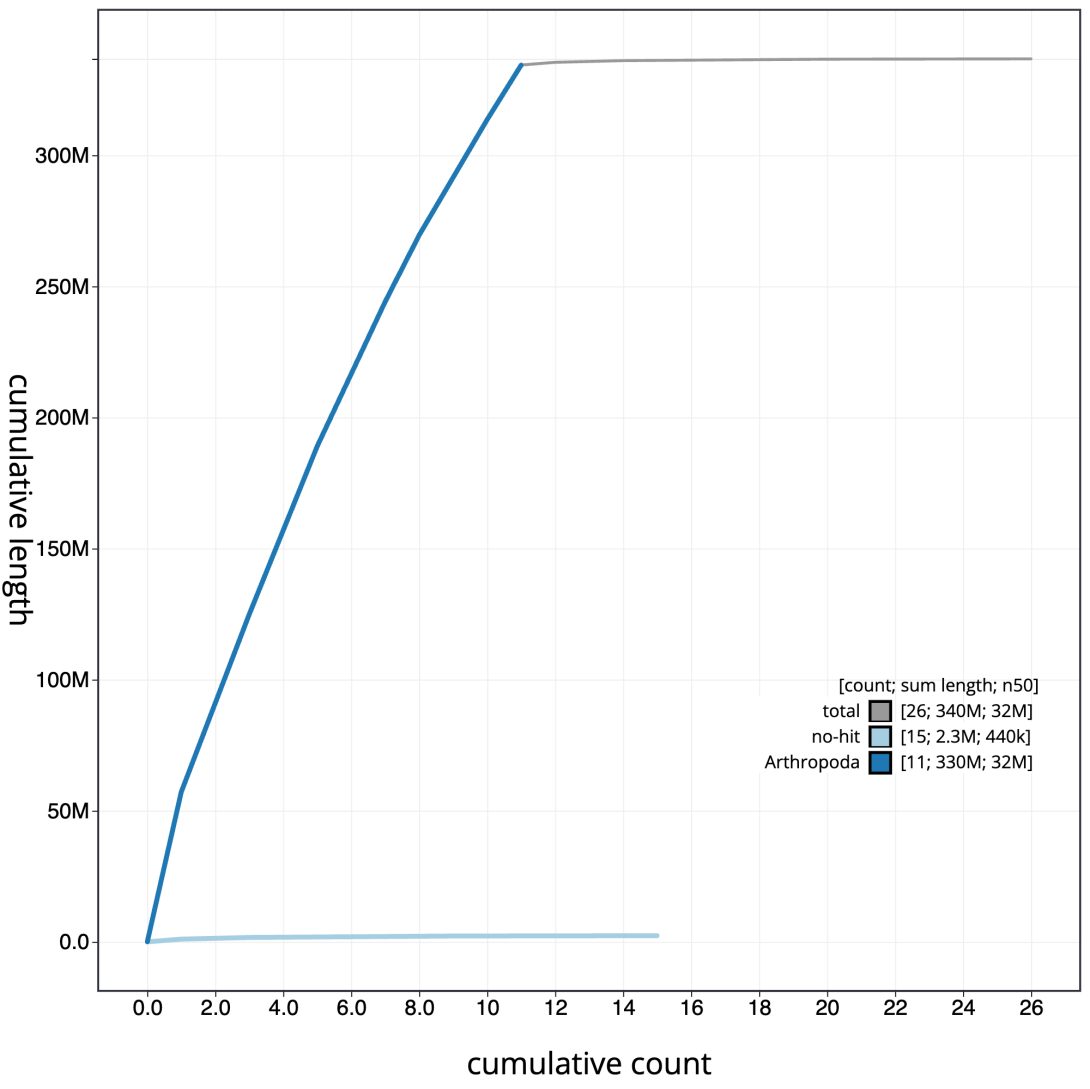
Genome assembly of *Lagria hirta*, icLagHirt1.1: BlobToolKit cumulative sequence plot. The grey line shows cumulative length for all scaffolds. Coloured lines show cumulative lengths of scaffolds assigned to each phylum using the buscogenes taxrule. An interactive version of this figure is available at https://blobtoolkit.genomehubs.org/view/Lagria%20hirta/dataset/CANAHW01/cumulative.

**Figure 5.**
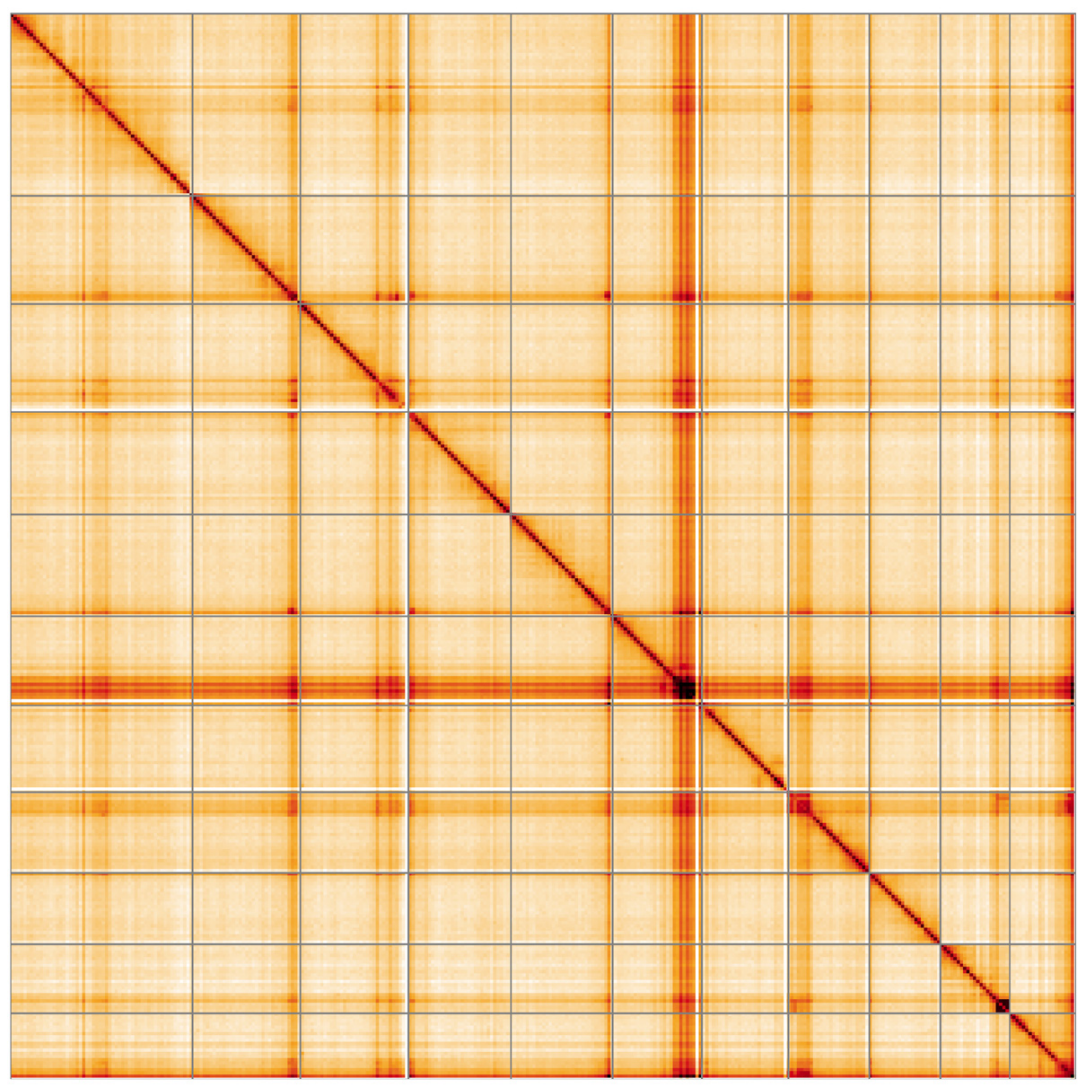
Genome assembly of *Lagria hirta*, icLagHirt1.1: Hi-C contact map of the icLagHirt1.1 assembly, visualised using HiGlass. Chromosomes are shown in order of size from left to right and top to bottom. An interactive version of this figure may be viewed at https://genome-note-higlass.tol.sanger.ac.uk/l/?d=WQn2sjdzQb6h70_XxZBwKQ.

The final assembly has a total length of 336.8 Mb in 25 sequence scaffolds with a scaffold N50 of 31.9 Mb ( Table 1). The snailplot in Figure 2 provides a summary of the assembly statistics, while the distribution of assembly scaffolds on GC proportion and coverage is shown in Figure 3. The cumulative assembly plot in Figure 4 shows curves for subsets of scaffolds assigned to different phyla. Most (99.73%) of the assembly sequence was assigned to 12 chromosomal-level scaffolds, representing 10 autosomes and the X and Y sex chromosomes. Chromosome-scale scaffolds confirmed by the Hi-C data are named in order of size ( Figure 5; Table 2). While not fully phased, the assembly deposited is of one haplotype. Contigs corresponding to the second haplotype have also been deposited. The mitochondrial genome was also assembled and can be found as a contig within the multifasta file of the genome submission.

**Table 1.** Genome data for *Lagria hirta*, icLagHirt1.1.

Project accession data		
Assembly identifier	icLagHirt1.1	
Species	*Lagria hirta*	
Specimen	icLagHirt1	
NCBI taxonomy ID	296003	
BioProject	PRJEB55604	
BioSample ID	SAMEA11025111	
Isolate information	icLagHirt1, male: whole organism (DNA sequencing) icLagHirt2: whole organism (Hi-C sequencing) icLagHirt3, female: head and thorax (RNA sequencing)	
Assembly metrics *		*Benchmark*
Consensus quality (QV)	67	*≥ 50*
*k*-mer completeness	100%	*≥ 95%*
BUSCO **	C:98.0%[S:97.1%,D:0.9%],F:0.1%, M:1.9%,n:2,124	*C ≥ 95%*
Percentage of assembly mapped to chromosomes	99.73%	*≥ 95%*
Sex chromosomes	X and Y chromosomes	*localised homologous pairs*
Organelles	mitochondrial genome assembled	*complete single alleles*
Raw data accessions		
PacificBiosciences SEQUEL II	ERR10144334	
Hi-C Illumina	ERR10123721	
PolyA RNA-Seq Illumina	ERR11641097	
Genome assembly		
Assembly accession	GCA_947359425.1	
*Accession of alternate haplotype*	GCA_947359435.1	
Span (Mb)	336.8	
Number of contigs	138	
Contig N50 length (Mb)	7.7	
Number of scaffolds	25	
Scaffold N50 length (Mb)	31.9	
Longest scaffold (Mb)	57.2	
Genome annotation		
Number of protein-coding genes	12,850	
Number of non-coding genes	1,541	
Number of gene transcripts	21,030	

* Assembly metric benchmarks are adapted from column VGP-2020 of “Table 1: Proposed standards and metrics for defining genome assembly quality” from ( Rhie *et al.*, 2021). ** BUSCO scores based on the endopterygota_odb10 BUSCO set using v5.3.2. C = complete [S = single copy, D = duplicated], F = fragmented, M = missing, n = number of orthologues in comparison. A full set of BUSCO scores is available at https://blobtoolkit.genomehubs.org/view/Lagria%20hirta/dataset/CANAHW01/busco.

**Table 2.** Chromosomal pseudomolecules in the genome assembly of *Lagria hirta*, icLagHirt1.

INSDC accession	Chromosome	Length (Mb)	GC%
OX375794.1	1	57.19	29.0
OX375795.1	2	33.89	29.5
OX375796.1	3	33.87	30.0
OX375797.1	4	32.2	29.5
OX375798.1	5	31.91	29.5
OX375800.1	6	27.17	29.0
OX375801.1	7	25.37	30.5
OX375802.1	8	22.34	29.0
OX375803.1	9	21.72	29.0
OX375804.1	10	20.79	29.0
OX375799.1	X	27.99	30.5
OX375805.1	Y	1.03	33.0
OX375806.1	MT	0.02	20.5

The estimated Quality Value (QV) of the final assembly is 67 with *k*-mer completeness of 100%, and the assembly has a BUSCO v5.3.2 completeness of 98.0% (single = 97.1%, duplicated = 0.9%), using the endopterygota_odb10 reference set ( *n* = 2,124).

Metadata for specimens, spectral estimates, sequencing runs, contaminants and pre-curation assembly statistics can be found at https://links.tol.sanger.ac.uk/species/296003.

## Genome annotation report

The *Lagria hirta* genome assembly (GCA_947359425.1) was annotated using the Ensembl rapid annotation pipeline ( Table 1; https://rapid.ensembl.org/Lagria_hirta_GCA_947359425.1/Info/Index). The resulting annotation includes 21,030 transcribed mRNAs from 12,850 protein-coding and 1,541 non-coding genes.

## Methods

### Sample acquisition and nucleic acid extraction

*Lagria hirta* specimens collected from Penhale Dunes, Cornwall, England, UK (latitude 50.37, longitude –5.14) on 2021-06-30 were used in this study. The specimen used for DNA sequencing was a male (specimen ID NHMUK014537045, ToLID icLagHirt1), while the specimen used for Hi-C was specimen ID NHMUK014537046 (ToLID icLagHirt2). The specimens were collected by Olga Sivell (Natural History Museum) using an aerial net. The specimens were identified by Brian Levey (National Museums Galleries of Wales) and dry frozen at –80°C.

The female specimen used for RNA sequencing (specimen ID NHMUK014439765, ToLID icLagHirt3), was collected from Fulham Green, London (latitude 51.47, longitude –0.18) on 021-08-04. This specimen was collected and identified by Maxwell Barclay (Natural History Museum) and then dry frozen at –80°C.

DNA was extracted at the Tree of Life laboratory, Wellcome Sanger Institute (WSI). The icLagHirt1 sample was weighed and dissected on dry ice with tissue set aside for Hi-C sequencing. Tissue from the whole organism was disrupted using a Nippi Powermasher fitted with a BioMasher pestle. High molecular weight (HMW) DNA was extracted using the Qiagen MagAttract HMW DNA extraction kit. HMW DNA was sheared into an average fragment size of 12–20 kb in a Megaruptor 3 system with speed setting 30. Sheared DNA was purified by solid-phase reversible immobilisation using AMPure PB beads with a 1.8X ratio of beads to sample to remove the shorter fragments and concentrate the DNA sample. The concentration of the sheared and purified DNA was assessed using a Nanodrop spectrophotometer and Qubit Fluorometer and Qubit dsDNA High Sensitivity Assay kit. Fragment size distribution was evaluated by running the sample on the FemtoPulse system. 

RNA was extracted from head and thorax tissue of icLagHirt3 in the Tree of Life Laboratory at the WSI using TRIzol, according to the manufacturer’s instructions. RNA was then eluted in 50 μl RNAse-free water and its concentration assessed using a Nanodrop spectrophotometer and Qubit Fluorometer using the Qubit RNA Broad-Range (BR) Assay kit. Analysis of the integrity of the RNA was done using Agilent RNA 6000 Pico Kit and Eukaryotic Total RNA assay.

Protocols employed by the Tree of Life laboratory are publicly available on protocols.io: https://dx.doi.org/10.17504/protocols.io.8epv5xxy6g1b/v1.

### Sequencing

Pacific Biosciences HiFi circular consensus DNA sequencing libraries were constructed according to the manufacturers’ instructions. Poly(A) RNA-Seq libraries were constructed using the NEB Ultra II RNA Library Prep kit. DNA and RNA sequencing was performed by the Scientific Operations core at the WSI on Pacific Biosciences SEQUEL II (HiFi) and Illumina NovaSeq 6000 (RNA-Seq) instruments. Hi-C data were also generated from whole organism tissue of icLagHirt2 using the Arima2 kit and sequenced on the Illumina NovaSeq 6000 instrument.

### Genome assembly, curation and evaluation

Assembly was carried out with Hifiasm ( Cheng *et al.*, 2021) and haplotypic duplication was identified and removed with purge_dups ( Guan *et al.*, 2020). The assembly was then scaffolded with Hi-C data ( Rao *et al.*, 2014) using YaHS ( Zhou *et al.*, 2023). The assembly was checked for contamination and corrected using the gEVAL system ( Chow *et al.*, 2016) as described previously ( Howe *et al.*, 2021). Manual curation was performed using gEVAL, HiGlass ( Kerpedjiev *et al.*, 2018) and Pretext ( Harry, 2022). The mitochondrial genome was assembled using MitoHiFi ( Uliano-Silva *et al.*, 2023), which runs MitoFinder ( Allio *et al.*, 2020) or MITOS ( Bernt *et al.*, 2013) and uses these annotations to select the final mitochondrial contig and to ensure the general quality of the sequence.

A Hi-C map for the final assembly was produced using bwa-mem2 ( Vasimuddin *et al.*, 2019) in the Cooler file format ( Abdennur & Mirny, 2020). To assess the assembly metrics, the *k*-mer completeness and QV consensus quality values were calculated in Merqury ( Rhie *et al.*, 2020). This work was done using Nextflow ( Di Tommaso *et al.*, 2017) DSL2 pipelines “sanger-tol/readmapping” ( Surana *et al.*, 2023a) and “sanger-tol/genomenote” ( Surana *et al.*, 2023b). The genome was analysed within the BlobToolKit environment ( Challis *et al.*, 2020) and BUSCO scores ( Manni *et al.*, 2021; Simão *et al.*, 2015) were calculated.

Table 3 contains a list of relevant software tool versions and sources.

**Table 3.** Software tools: versions and sources.

Software tool	Version	Source
BlobToolKit	4.1.5	https://github.com/blobtoolkit/blobtoolkit
BUSCO	5.3.2	https://gitlab.com/ezlab/busco
Hifiasm	0.16.1-r375	https://github.com/chhylp123/hifiasm
HiGlass	1.11.6	https://github.com/higlass/higlass
Merqury	MerquryFK	https://github.com/thegenemyers/MERQURY.FK
MitoHiFi	2	https://github.com/marcelauliano/MitoHiFi
PretextView	0.2	https://github.com/wtsi-hpag/PretextView
purge_dups	1.2.3	https://github.com/dfguan/purge_dups
sanger-tol/genomenote	v1.0	https://github.com/sanger-tol/genomenote
sanger-tol/readmapping	1.1.0	https://github.com/sanger-tol/readmapping/tree/1.1.0
YaHS	yahs-1.1.91eebc2	https://github.com/c-zhou/yahs

### Genome annotation

The Ensembl gene annotation system ( Aken *et al.*, 2016) was used to generate annotation for the *Lagria hirta* assembly (GCA_947359425.1). Annotation was created primarily through alignment of transcriptomic data to the genome, with gap filling via protein-to-genome alignments of a select set of proteins from UniProt ( UniProt Consortium, 2019).

### Wellcome Sanger Institute – Legal and Governance

The materials that have contributed to this genome note have been supplied by a Darwin Tree of Life Partner. The submission of materials by a Darwin Tree of Life Partner is subject to the **‘Darwin Tree of Life Project Sampling Code of Practice’**, which can be found in full on the Darwin Tree of Life website here. By agreeing with and signing up to the Sampling Code of Practice, the Darwin Tree of Life Partner agrees they will meet the legal and ethical requirements and standards set out within this document in respect of all samples acquired for, and supplied to, the Darwin Tree of Life Project. 

Further, the Wellcome Sanger Institute employs a process whereby due diligence is carried out proportionate to the nature of the materials themselves, and the circumstances under which they have been/are to be collected and provided for use. The purpose of this is to address and mitigate any potential legal and/or ethical implications of receipt and use of the materials as part of the research project, and to ensure that in doing so we align with best practice wherever possible. The overarching areas of consideration are:

- Ethical review of provenance and sourcing of the material
- Legality of collection, transfer and use (national and international)

Each transfer of samples is further undertaken according to a Research Collaboration Agreement or Material Transfer Agreement entered into by the Darwin Tree of Life Partner, Genome Research Limited (operating as the Wellcome Sanger Institute), and in some circumstances other Darwin Tree of Life collaborators.

## Data Availability

European Nucleotide Archive: *Lagria hirta*. Accession number PRJEB55604; https://identifiers.org/ena.embl/PRJEB55604 ( Wellcome Sanger Institute, 2022). The genome sequence is released openly for reuse. The *Lagria hirta* genome sequencing initiative is part of the Darwin Tree of Life (DToL) project. All raw sequence data and the assembly have been deposited in INSDC databases. Raw data and assembly accession identifiers are reported in Table 1.

## Author information

Members of the Natural History Museum Genome Acquisition Lab are listed here: https://doi.org/10.5281/zenodo.4790042.

Members of the Darwin Tree of Life Barcoding collective are listed here: https://doi.org/10.5281/zenodo.4893703.

Members of the Wellcome Sanger Institute Tree of Life programme are listed here: https://doi.org/10.5281/zenodo.4783585.

Members of Wellcome Sanger Institute Scientific Operations: DNA Pipelines collective are listed here: https://doi.org/10.5281/zenodo.4790455.

Members of the Tree of Life Core Informatics collective are listed here: https://doi.org/10.5281/zenodo.5013541.

Members of the Darwin Tree of Life Consortium are listed here: https://doi.org/10.5281/zenodo.4783558.
