# Supplementary material for: The genome sequence of a darkling beetle, Lagria hirta (Linnaeus, 1758)
Source: Wellcome Open Res. 2023 Nov 1;8:501. [Version 1] doi: 10.12688/wellcomeopenres.20232.1 (PMC10905005; doi:10.12688/wellcomeopenres.20232.1)
Supplement: Peer review history

# Reviewer report: Reviewer response for version 1

**DOI:** 10.21956/wellcomeopenres.22404.r74575

**Published:** 2024-02-29

**Authors:** Manee M Manee

**Affiliations:** 1 King Abdulaziz City for Science and Technology, Riyadh, Saudi Arabia

The manuscript titled "The Genome Sequence of a Darkling Beetle, *Lagria hirta*," presents a detailed account of the genomic analysis of *Lagria hirta*, including the assembly and annotation of its genome. Here are specific areas for improvement:

 - Enhancing the background with a detailed phylogenetic positioning of *Lagria hirta* within the Tenebrionidae family, alongside a comprehensive comparative genomic analysis with its close relatives and other family members, could significantly enrich the context. This approach would illuminate evolutionary adaptations, gene family dynamics, and unique genomic features, offering a deeper understanding of Lagria hirta's significance and its comparative evolutionary biology within the beetle order.

 - To enhance methodological clarity, detail the bioinformatics workflows, specify parameter settings for genome assembly and annotation, and justify the selections of tools. Include criteria for software choices, details of custom scripts, and their impact on result accuracy and reproducibility.

Are sufficient details of methods and materials provided to allow replication by others?

Partly

Is the rationale for creating the dataset(s) clearly described?

Yes

Are the datasets clearly presented in a useable and accessible format?

Yes

Are the protocols appropriate and is the work technically sound?

Partly

Reviewer Expertise:

Bioinformatics, genomics, evolutionary biology

I confirm that I have read this submission and believe that I have an appropriate level of expertise to confirm that it is of an acceptable scientific standard, however I have significant reservations, as outlined above.

# Reviewer report: Reviewer response for version 1

**DOI:** 10.21956/wellcomeopenres.22404.r71831

**Published:** 2024-02-20

**Authors:** Reinhard Predel

**Affiliations:** 1 University of Cologne, Cologne, Germany

The genome sequence of *Lagria hirta* is provided, the assembly is of very good quality. It is the first genome of a species from the subfamily Lagriinae, transcriptomes of that species have been previously published. The background provided is restricted to some easily accessible information on morphology and natural history.  I miss some more detailed information about the phylogenetic position of the Lagriinae within the hyperdiverse Tenebrionidae. The first beetle whose genome was sequenced, *Tribolium castaneum*, also belongs to Tenebrionidae, and the genome of this species is now one of the most intensively studied in insects. I think this could be important background information, the report would also benefit from some comparative information, e.g. about genome size of *Tribolium* compared to *Lagria*, etc.

Are sufficient details of methods and materials provided to allow replication by others?

Yes

Is the rationale for creating the dataset(s) clearly described?

Yes

Are the datasets clearly presented in a useable and accessible format?

Yes

Are the protocols appropriate and is the work technically sound?

Yes

Reviewer Expertise:

evolution of insects, proteomics, transcriptomics

I confirm that I have read this submission and believe that I have an appropriate level of expertise to confirm that it is of an acceptable scientific standard.

# Reviewer report: Reviewer response for version 1

**DOI:** 10.21956/wellcomeopenres.22404.r69679

**Published:** 2023-11-20

**Authors:** Yi-Ming Weng

**Affiliations:** 1 Department of Entomology, University of Wisconsin Madison, Madison, WI, USA

The genome assembly of the darkling beetle by Sivell *et al.*, is reviewed. The report contains sufficient details in describing the general biology, morphology, and genomic feature of this species. The assembly is very complete and well represented and the gene annotation process also includes the transcriptomic information, demonstrating it's high data quality. This will be a good resource for many future studies.

Are sufficient details of methods and materials provided to allow replication by others?

Yes

Is the rationale for creating the dataset(s) clearly described?

Yes

Are the datasets clearly presented in a useable and accessible format?

Yes

Are the protocols appropriate and is the work technically sound?

Yes

Reviewer Expertise:

Entomology, population genetics, evolutionary genomics

I confirm that I have read this submission and believe that I have an appropriate level of expertise to confirm that it is of an acceptable scientific standard.
